# Screening for Human Papillomavirus in a Low- and Middle-Income Country

**DOI:** 10.1200/JGO.18.00233

**Published:** 2019-05-03

**Authors:** Aaron E. Atkinson, Carlos Alberto Matute Mandujano, Suyapa Bejarano, Linda S. Kennedy, Gregory J. Tsongalis

**Affiliations:** ^1^ Dartmouth Hitchcock Health System, Lebanon, NH; ^2^ Geisel School of Medicine at Dartmouth, Hanover, NH; ^3^ Universidad Católica Honduras, San Pedro Sula, Honduras; ^4^ Liga Contra el Cáncer, San Pedro Sula, Honduras

## Abstract

**PURPOSE:**

Low- and middle-income countries have high incidences of cervical cancer linked to human papillomavirus (HPV), and without resources for cancer screenings these countries bear 85% of all cervical cancer cases. To address some of these needs, brigade-style screening combined with sensitive polymerase chain reaction–based HPV testing to detect common high-risk HPV genotypes may be necessary.

**METHODS:**

We deployed an inexpensive DNA extraction technique and a real-time polymerase chain reaction–based HPV genotyping assay, as well as Papanicolaou testing, in a factory in San Pedro Sula, Honduras, where 1,732 women were screened for cervical cancer.

**RESULTS:**

We found that 28% of participants were positive for high-risk HPV, with 26% of HPV-positive participants having more than one HPV infection. Moreover, the most common HPV genotypes detected were different than those routinely found in the United States.

**CONCLUSION:**

This work demonstrates a deployable protocol for HPV screening in low- and middle-income countries with limited resources to perform cytopathology assessment of Pap smears.

## INTRODUCTION

Cervical cancer in low- and middle-income countries (LMICs) accounts for 85% of an estimated 528,000 new cases globally and 266,000 deaths annually.^1-3^ In many LMICs, cervical cancer is the leading cause of cancer-related mortality and attributable to limited screening programs. A lack of funding, trained cytopathologists to review Papanicolaou test slides, and other health care providers for follow-up care that comprise the needed infrastructure does not allow for routine cervical cancer screening. To alleviate some of these burdens associated with cervical cancer screening, we developed and validated a process with which to screen women using a simple, high-risk human papillomavirus (hrHPV) DNA test.^4^ We demonstrate that DNA testing for hrHPV using crude cell lysates and real-time polymerase chain reaction (PCR) with lyophilized reagents was feasible to perform in a mobile system so that women in various regions of Honduras could be tested.^5^

**CONTEXT**

**Key Objective**
Could high-risk HPV genotyping be implemented in a low- and middle-income country setting for screening purposes?
**Knowledge Generated**
Using modified DNA extraction methods and lyophilized reagents for real-time polymerase chain reaction, we were implemented screening of more than 1,000 participants. The prevalence of high-risk human papillomavirus types was different than that observed in high-income countries.
**Relevance**
This approach can help to alleviate the limitations associated with cervical cancer screening in low- and middle-income countries.

As the causative agent for cervical cancer, detection of hrHPV types, which is less subjective, has the potential to improve outcomes relative to less-sensitive visual or cytologic tests.^6-11^ Several low-cost HPV screening tests have been developed specifically for use in LMICs. Two of these tests, however, cannot distinguish between HPV types or resolve coinfections known to have higher rates of cervical disease, whereas a third less-sensitive immunologic test only detects HPV types 16, 18, and 45.^12-15^ Cervical cancer screening guidelines have recently been published that recommend DNA testing for the detection of hrHPV in a liquid cytology sample as a sole test or in conjunction with Papanicolaou test.^16^ Use of instrumentation and assays that are US Food and Drug Administration approved in the United States is not feasible, nor sustainable, in Honduras as a result of cost and facility logistics. We therefore developed a low-cost, simple DNA testing method for hrHPV using a crude cell lysate and real-time PCR instrument.^4,5^ In several studies, we showed that hrHPV type distributions were more reflective of those found in Asia than in North America.^4,5,17^ This geographically unexpected distribution underscores the need for HPV genotype surveillance to effectively triage hrHPV-positive participants for follow-up care and to strategize the implementation of successful vaccination programs.^18^

In the current study, we determine the hrHPV type frequency in women working in a manufacturing facility in San Pedro Sula, Honduras.

## METHODS

This study was approved by the Dartmouth Committee for the Protection of Human Subjects and the Universidad de Catolica in San Pedro Sula, Honduras. Cervical samples were collected using cervical brushes on 1,732 participants who were employees at a manufacturing site in San Pedro Sula, Honduras, for Papanicolaou test and hrHPV testing. Samples were collected by trained physicians and medical students. Participants with a positive hrHPV result underwent a follow-up examination at a local cancer center.

**FIG 1:**
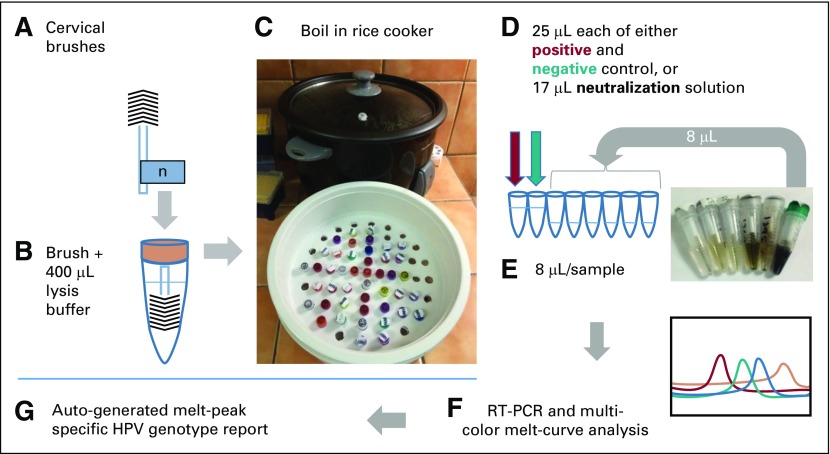
Flowchart of rice cooker boiling alkaline lysis DNA extraction. (A) Cervical brushes were obtained and accessioned. (B) Brushes were cut to fit a tube that contained 400 μL lysis buffer. (C) Tubes were placed in the colander of a rice cooker and boiled for 10 minutes. (D) Twenty-five microliters of the supplied and resuspended positive and negative controls were added to two reaction tubes and 17 μL of 70 mM Tris pH 8.0 was added to the remaining reaction tubes. (E) Eight microliters of each sample was added to reaction tubes for a final volume of 25 μL. (F) Real-time polymerase chain reaction (RT-PCR) and multicolor melt-curve analysis were performed. (G) A report was compiled from the results provided by the instrument software. HPV, human papillomavirus; n, patient accession number.

All cervical brushes and corresponding Papanicolaou test slides were assigned a unique study identification number (Fig 1A). After slide prep, brushes were misted with ethanol and air dried before being individually packaged and stored at room temperature for 30 to 40 days before processing. Cell lysates from cervical swabs were made as previously described with several modifications.^4^ In brief, dried cervical brushes were cut and placed inside individual tubes that contained 400 µL of cell lysis buffer consisting of 50 mM NaOH and 0.2 μM EDTA in nuclease-free water (Fig 1B). Tubes were boiled for 10 minutes using an inexpensive locally sourced rice cooker, the plastic colander of which had been bored out to accommodate the 1.5-mL screw-cap tubes (Fig 1C). Next, 17 μL of 70 mM Tris pH 8.0 was added to rehydrate the assay tubes that contained lyophilized MeltPro high-risk genotyping reagents (QuanDx/Zeesan Biotech, San Jose, CA). Finally, 8 μL of cell lysate was added to the rehydrated reaction tubes for a final volume of 25 μL. Separately, 25 μL of each positive and negative control were pipetted into lyophilized reagent tubes (Figs 1D and 1E). Rehydrated reagents with cell lysate were then mixed and loaded directly onto the SLAN-96 real-time PCR instrument (QuanDx/Zeesan Biotech) and run using the SLAN 8.2.2 software per the manufacturer’s protocol. HPV typing results were available within 2.5 hours (Figs 1F and 1G).

This assay can detect and distinguish 14 hrHPV types (HPV types 16, 18, 31, 33, 35, 39, 45, 51, 52, 56, 58, 59, 66, and 68) in addition to an internal human DNA sequence control. This is accomplished through a multiplexed PCR with end point melt-curve analysis that resolves type-specific melting temperatures for four distinct probes labeled with ROX (HPV 31, 33, 16, 35, 68, and 18), CY5 (HPV 56, 52, 45, and 39), FAM (HPV 59, 66, 58, and 51), and HEX for the internal control (Table 1). Sample results using the system were negative, HPV positive by type, or invalid as a result of a failed reaction that did not generate either signal for the internal control or HPV. Invalid samples were collected and stored until repeat reactions could be performed after the addition of 20 μL of 1 M Tris pH 8.0. Each kit is supplied with both lyophilized reagents and controls which did not require refrigeration during the course of these experiments.

**TABLE 1:**
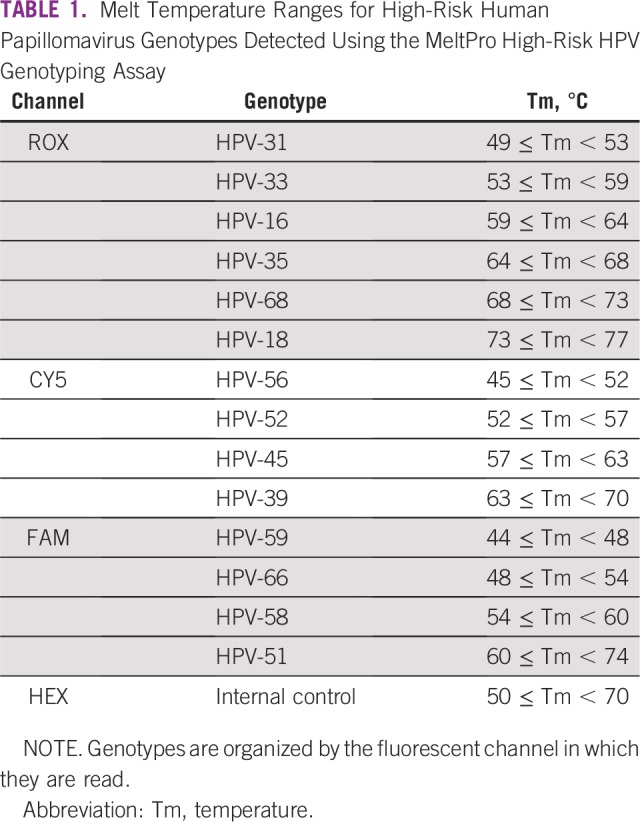
Melt Temperature Ranges for High-Risk Human Papillomavirus Genotypes Detected Using the MeltPro High-Risk HPV Genotyping Assay

## RESULTS

**FIG 2:**
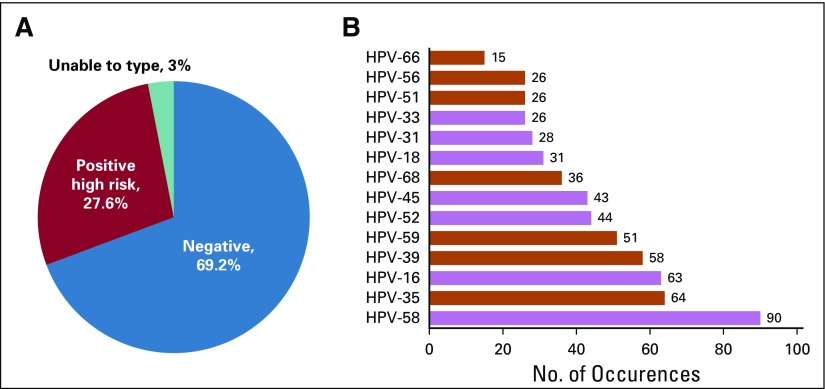
Summary of human papillomavirus (HPV) infections. (A) Distribution of HPV infections among the 1,732 tested samples. (B) Genotype distribution among all 480 HPV-positive samples. Genotypes of the HPV strains present in the nonavalent vaccine are shown in black, whereas those strains not included in the vaccine are shown in gray. The total number of occurrences is listed above each histogram.

Of 1,732 samples, 480 (28%) were positive for high-risk HPV, and 1,199 samples (69%) had no detectable HPV. Fifty-three samples (3%) failed to amplify either the internal control or an HPV target and were deemed to be invalid and subsequently unable to type (Fig 2A). HPVs 58, 35, and 16 were the most common genotypes present with 90 (19% of positive samples), 64 (13% of positive samples), and 63 (13% of positive samples) infections, respectively (Fig 2B). Of note, there were only 31 HPV 18–positive samples.

**FIG 3:**
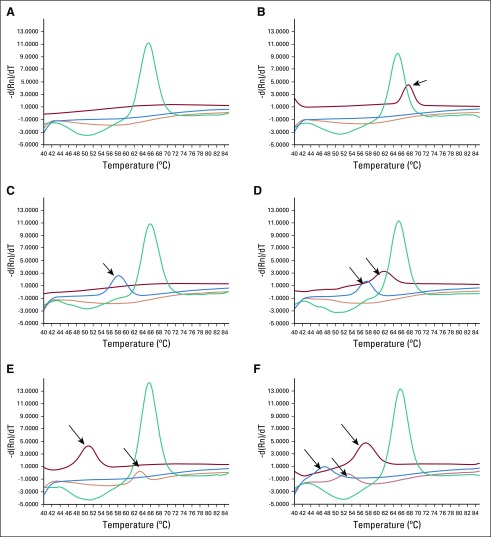
Examples of melt-peak outputs. (A) Negative. (B) Human papillomavirus (HPV) -39 positive (arrow). (C) HPV-58 (arrow). (D) HPV-58, HPV-45 coinfection (arrows). (E) HPV-16, HPV-56 coinfection (arrows). (F) HPV-31, HPV-52, HPV-59 coinfection (arrows). Internal control (green/Hex); HPVs 31, 33, 16, 35, 68, 18 (orange/ROX); HPVs 56, 52, 45, 39 (red/CY5); HPVs 59, 66, 58, 51 (blue/FAM).

Among 480 HPV-positive samples, 126 (26%) were positive for two or more HPV genotypes. There were five participants with HPV 58/HPV 16 coinfections and a single participant with an HPV 58/HPV 35 coinfection. No HPV 16/HPV 35 or HPV 16/HPV 18 coinfections were identified. Individual results of the identified HPV infections are shown in Figure 3.

## DISCUSSION

Cervical cancer screening strategies, including cytologic and HPV screens designed for higher-income countries with widespread health care access and reliable follow-up care, are difficult to implement in LMICs where those resources are scarce. Cytologic examination in LMICs is costly and the number of cytologists available is not sufficient to perform the analyses, especially in countries in which gaps in health care access are prevalent. Resolving these problems requires a paradigm shift. Low-cost HPV screening techniques offer a potential solution as there is now strong evidence to show that HPV-based screening assays are not only a more sensitive means with which to detect underlying cervical abnormalities compared with conventional cytology,^10,11,19,20^ but can also be performed by nontechnical staff. Moreover, higher negative predictive values with PCR-based HPV tests offer longer reassurance to patients that they are not at substantial risk of developing cervical cancer.^10,11,21-23^ In this context, DNA-based HPV screening methods are less subjective, more robust, and have been shown to have higher sensitivity^10,11,23^ and the ability to distinguish between many coinfections that increase the risk of cervical disease.^10,11,15^

For the 480 participants (28%) we identified with HPV infection, the benefits of screening will, we hope, translate into earlier interventions and a reduction in overall cervical cancer rates upon referral for cytologic examination and follow-up. For the remaining 1,202 HPV-negative participants (69%), there is strong confidence that they are relatively safe from developing cervical cancer for the next 5 years.^21-24^ However, continued and diligent HPV screening is critical for sustained impact and effectiveness of cervical cancer prevention.^25,26^ High-risk HPV screening should include data on vaccinations and genotypic information for both infection prevalence and HPV-related cancers.^19^ Such screening programs already have documented suppression of HPVs 16 and 18 in response to the bivalent vaccine.^27^ In other studies, particularly those in areas with prevaccine HPV genotype distributions that are known to include genotypes not included in vaccines, such as HPV 58, controversial active surveillance is underway to closely monitor for the potential of type replacement after widespread use of the bivalent vaccine.^28,29^

In the current study, we found no HPV 16 and HPV 18 coinfections and relatively few single HPV 16 or HPV 18 infections; therefore, direct coverage provided by the bivalent vaccine against infection and possible progression to cervical cancer would equate to approximately 14% among those that are HPV positive in this study. When considering the nonavalent vaccine that covers HPVs 6,11, 16, 18, 31, 33, 45, 52, and 58, approximately 47% of those that are HPV positive in this study could receive some amount of protection from either single or coinfections and possible progression to cervical cancer among those genotypes. However, approximately 53% of participants in the current study would not have benefited from the nonavalent vaccine on the basis of genotype prevalence alone (Fig 2B). There may be some cross-reactive protection against HPV 31 and potentially HPVs 33 and 45, although data on any bivalent vaccine cross-protection are mixed.^17,27,30^ In this study of factory workers in San Pedro Sula, Honduras, our results show a prevalence of HPV 58 and HPV 35 infections, with 90 (19%) and 64 (13%) of 480 infections, respectively (Fig 2B). Neither of these genotypes would be covered by the Honduran bivalent vaccination program. In addition, with 26% of HPV-positive participants having more than one detectable HPV genotype, our data underscore the benefit of HPV screening that is able to differentiate coinfection hrHPV types for epidemiologic purposes, as these participants are at the greatest risk for cervical cancer.^15,31^

Global HPV genotype distribution is broadly known to have geographic differences that must be considered for cervical cancer prevention and surveillance strategies. Our previous work found a high prevalence of HPV 52 in an isolated Honduran community located in the largest wilderness tract in Central America.^5^ HPV 52 was also found in neoplastic lesions at a prevalence that was higher than expected in Mexico.^32^ Similarly, HPV 52 and 58 are known to have a higher distribution in Asia relative to other regions.^28,33^ Not surprisingly, subsequent studies found that HPV 58 accounts for a larger proportion of cervical cancer burden in Asia than elsewhere.^34,35^

The HPV distribution identified in this participant population and limited number of in-country pathologists makes the use of primary HPV testing, as described here, both critical and effective, especially given the interim clinical guidance for the use of such testing for cervical cancer screening.^31^ Triaging patients on the basis of positive HPV status within these guidelines not only allows for the most effective use of the limited number of pathologists, but the HPV genotypic information provides critical information for both patient care and to support vaccination strategies adopted in those countries.^27^ A limitation of the current study is in the convenience sampling with respect to women working in a single factory in one Honduran city as well as the age of participants being limited to a working group.

**FIG 4:**
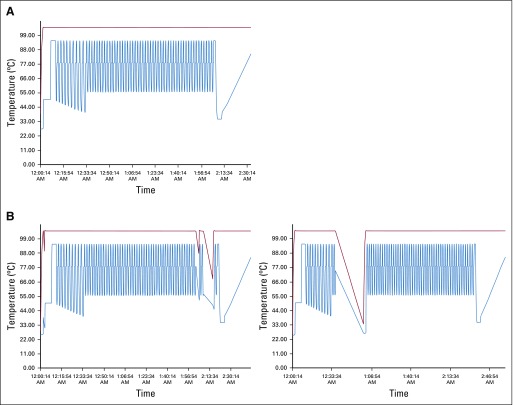
Instrument thermal profiles. (A) Reaction proceeding without power interruption and (B) with various interruptions to instrument power. Thermal cycler lid temperature (red); block temperature (blue).

Last, the low-cost DNA lysate and sensitive HPV assay described here allowed us to process 94 samples in less than 2.5 hours and return rapid genotype-specific results. This approach offers the following benefits: in contrast to other PCR-based assays that use hydrolysis probes, the equipment can be run off of a generator or variable power sources, as the system returns results despite power interruptions (Fig 4); the PCR system itself uses LEDs that require little, if any, maintenance; the DNA extraction method uses minimal and readily available disposables; lyophilized reagents were tested without refrigeration for 2 weeks and maintained their efficacy^4^; and the combination of lyophilized reagents, crude lysate, and HPV genotyping performed by the instrument makes for an easy-to-perform operation that is suitable for testing in locations without highly trained personnel. These features allow for near-patient care diagnosis that is of critical importance for combining screening and treatment strategies in LMICs.
